# The Book World of Medicine and Science

**Published:** 1904-01-23

**Authors:** 


### The Book World of Medicine and Science.

The Exact Science of Health Based upon Life's Great Law. By Robert Walter, M.D. Vol. I.: Principles. (London: Kegan Paul, Trench, Trübner and Co.)

The writer of this book conceives himself to have discovered a truth not less important than the law of gravitation. This he names "Life's Great Law." It is the outcome, he tells us, of "twenty years of study and reflection." In its practical value to mankind it "exceeds all other forms of human knowledge." And the discoveries associated with it yield a basis for a "reasonable philosophy of existence consistent with all known truth." These terms, be it observed, are not phrases expressing appreciation of the triumphs of Francis Bacon or Charles Darwin. They are descriptions by the author (who, of course, must know) of the work which, in the form of a book, Dr. Robert Walter, of Walter's Park, Pennsylvania, U.S.A., now sends forth "on an errand of mercy to suffering millions." In our own opinion the book is a verbose, wearisome, and pretentious jumble of copy-book platitudes and pseudo-scientific nonsense. We find in it no redeeming feature. It is difficult, indeed, to imagine the methods of study and the nature of the reflections which, pursued during a period of twenty years, can have produced a result so empty alike of value and interest. The much vaunted "Law" is nothing more than the old doctrine of vital force with the addition of an absurd proposition to the effect that the success of the operation of the force is "inversely to the degree of its activity." The author finds a parallel for this in the law of gravitation, though he admits that "the phraseology is somewhat different." But he has no doubt that "inversely to the square of the distance . . . really means . . . inversely to the degree of activity of the force.' That such a conviction could be entertained by any vertebrate animal we would, short of a personal assurance, never have believed. But Dr. Walter claims the distinction, and we readily recognise his right to entertain whatever nonsense he deems suitable to the quality of his mind. The settled conviction that "if Newton lived to-day" he would withdraw his own formula in favour of a suggestion emanating from Walter's Park is another article in our author's creed. It is hopeless to expect that any shaft of criticism can penetrate so abundant a self-complacency. Before the days of Dr. Walter there was a great philosopher who valued philosophy largely for what he called "fruit," and on the importance of practical results Dr. Walter also is himself insistent. The main catalogue of these is reserved for a second volume. But as a foretaste we are here supplied with a "sample treatment" of diphtheria. In this we read that kali mur 6 X is to be recommended "if the membrane be gray," and natrum sulph. 6 X "if it has a yellowish cast." Further illustrations of the same order may be confidently expected in Vol. II. To Vol. I. we now bid a cordial, affectionate, and highly-appreciative farewell; our good wishes and opinion being, like "Life's Great Law," inversely to the energy of their expression.

The Religious Sense in its Scientific Aspect. By Greville Macdonald, M.D. (London: Hodder and Stoughton. 1903.)

This book consists of three lectures given by Dr. Macdonald before students of the several departments at King's College. It may be defined as an attempt to present natural phenomena as parts of an ordered scheme and to find the element of moral purpose which it is suggested dominates the entire plan. The idea is worked out and illustrated in a very ingenious and beautiful fashion, and in the manner of its presentation there may be detected in no small measure evidences of both the insight and elevation of the poetic spirit Opinions will differ regarding the validity of some of the arguments advanced, but none will question the reverence for truth or the note of aspiration which mark the spirit and language of every chapter. For our own part we regard Dr. Macdonald's thesis as a necessary part of a true physiology and not less within the region of science than the study of blood-pressure or of the phenomena of digestion. The first introduction to the study of biology is so full of the pervading influence of mechanical and chemical explanations that the youthful mind is apt to conclude that the entire universe can be expressed in terms of the revolving cylinder or the chemical reaction, and there are lecture-rooms and laboratories in which, at all events by implication, this idea is emphasised. The spirit and doctrine of physiology are sometimes, we think, hidden by the wealth of apparatus intended to illustrate them. Hence we welcome this book because it insists upon the view that all, and not a part only, of the phenomena of life fall within the claim of scientific observation and argument, and because also its ethical spirit and inspiration are founded not on dogma and authority but on the secure basis of reason and moral purpose.

Dr. John Brown: A Biography and a Criticism. By the late John Taylor Brown, LL.D., F.S.A. (Scot.). Edited, with a short sketch of the Biographer, by W. B. Dunlop, M.A. (London: Adam and Charles Black. 1903.)

A pathetic interest attaches to the appearance of this book due to the death of the writer ere his manuscript had reached the printer's hands. Though Dr. John Brown, more than most authors, can be pictured from his writings, it is no slight advantage to have a sketch of his life from one associated with him for many years in the ties of intimate friendship. To those who know "Marjorie Fleming," and "Rab and His Friends," such a tribute of affection will be particularly welcome. For those who do not, if such there be, this biography of a vigorous and charming writer should prove an introduction to many pleasant hours. Medicine and literature on the other side of the Atlantic share a common glory in "The Autocrat of the Breakfast Table." We may reflect with no less satisfaction that the "Horœ Subsecivœ" and the essay on "Locke and Sydenham," are from the pen of a British practitioner.

Cancer: An Exhaustive Treatise and an Appeal ad Populum to Accept Nature's Own and Only Remedy. By Carlo Carillo, M.D., Ph.D. (Swan, Sonnenschein & Co.)

This book consists largely of statements which the writer has collected from various sources, together with some of the author's own ideas. It is ill-arranged and in most part farcical. We have been able to find no evidence, in the statements which he has put forward to support his views, which would have any weight with scientific observers, and therefore must decline to seriously consider the volume.

